# Cellular Senescence and Inflammaging in Age-Related Diseases

**DOI:** 10.1155/2018/9076485

**Published:** 2018-04-17

**Authors:** Fabiola Olivieri, Francesco Prattichizzo, Johannes Grillari, Carmela R. Balistreri

**Affiliations:** ^1^Department of Clinical and Molecular Sciences, Università Politecnica delle Marche, Ancona, Italy; ^2^Center of Clinical Pathology and Innovative Therapy, INRCA-IRCCS National Institute, Ancona, Italy; ^3^IRCCS MultiMedica, Milan, Italy; ^4^University of Natural Resources and Life Sciences Vienna, Vienna, Austria; ^5^Department of Pathobiology and Medical Biotechnologies, University of Palermo, Corso Tukory 211, 90134 Palermo, Italy

The term *cellular senescence* was used in 1961 for the first time by Hayflick and Moorhead [1], to define the mechanism determining the irreversible loss of the proliferative activity of human somatic cells [1]. In this respect, cellular senescence has been usually viewed as one of the regulatory mechanisms able to stop the eventual uncontrolled proliferation of old and injured cells, mediating an activity likely similar to a tumour suppressor gatekeeper [2]. Accordingly, oncogene overexpression (i.e., *H-ras*) in primary cells is sufficient to trigger cellular senescence, a phenomenon defined *oncogene-induced senescence* [2]. Indeed, negation or bypass of oncogene-induced senescence is considered an obligatory step to develop a tumour. Later, other groups discovered that cellular senescence also occurs in differentiated cells exposed to several stressors, mainly DNA-damaging agents such as prooxidant molecules, radiation, and chemotherapeutics [3–5]. Increasing evidence has highlighted various physiological roles of cellular senescence, consequently providing a putative evolutionary explanation for this biological phenomenon. For instance, in 2013, Muñoz-Espín et al. and Storer et al. groups underlined a crucial role for cellular senescence in mammalian embryonic development by studying mouse, chick, and human embryos [6, 7]. In this context, senescence promotes tissue remodeling and it seems to represent the “*father evolutional mechanism*” of *damage-induced senescence*. In 2014, Demaria et al.'s group [8] showed that senescent fibroblasts and endothelial cells appear very early in response to a cutaneous wound, where they accelerate wound closure by inducing myofibroblast differentiation through the secretion of platelet-derived growth factor AA (PDGF-AA). In addition, it has been also demonstrated that *forced cellular reprogramming* induces senescence, and senescent cells (SCs) create a permissive environment for reprogramming itself [9]. This evidence leads to an interesting hypothesis based on the concept that SCs act as a trigger of tissue remodeling, following a precise regulated succession of events, characterized by the arrest of their proliferative activity and release of mediators to recruit innate immune cells, consequently promoting tissue regeneration and their elimination. These steps may result uncompleted in old tissues or in pathological circumstances, determining the accumulation of SCs [9, 10]. Accordingly, an increased number of SCs characterizes the old tissues during aging and age-related diseases (ARDs), including cardiovascular diseases (CVDs), type 2 diabetes (T2DM), musculoskeletal disorders, various types of cancer, and neurodegenerative diseases. Thus, cellular senescence happens in both physiological (i.e., embryonic development, wound healing, and tumour suppression) and pathological processes (i.e., ARDs), by providing beneficial effects early in life and deleterious consequences later in life, in association with the accumulation of SCs in different tissues and organs. The different age-tailored effects played by SCs can be explained by the *theory of antagonistic pleiotropy* formulated by Williams [11]. This vision is corroborated by the observation that selective removal of SCs is sufficient to sensibly increase the lifespan of normal, genetically heterogeneous mice [12, 13]. These findings prompted the research of substances able to selectively promote the clearance of SCs, that is, senolytics (see below) [14, 15]. Some of these treatments have been demonstrated to delay the onset of ARDs and consequently to extend the healthspan (i.e., the length of time one lives in good health) in mice [15–18].

Despite the accumulating evidence regarding the contribution of SCs to the aged phenotype, the mechanisms through which SCs drive aging have not been fully elucidated, as well as their effective relationship with the major ARDs in humans. To this purpose, the lack of universal biomarkers for senescence *in vivo* has possibly contributed to hinder the detection of SCs in humans. However, growing evidence shows that SCs exert detrimental effects on the tissue microenvironment, generating pathological facilitators or aggravators (amply quoted in [19]). Accordingly, it has been suggested that SCs contribute to aging and ARD onset through the *senescence-associated secretory phenotype* (SASP), which consists in the secretion of a variety of soluble factors, such as proinflammatory mediators and matrix-degrading molecules [19]. SASP contributes to fuel a state of chronic, systemic, low-grade inflammation, called “*inflammaging*,” which is one of the main risk factors for the development of the major ARDs [19]. The rate of reaching the threshold of proinflammatory status over which diseases/disabilities ensue depends on a complex interaction between genetic, environmental, and stochastic factors [19]. Immune cells, especially macrophages, emerge as key players in the induction and maintenance of inflammaging [20, 21]. As a result, it might be hypothesized a typical phenomenon of “*macrophaging*,” which could contribute to reduce the clearance of SCs [19–22]. In turn, the age-related SC accumulation promotes immune system activation, and the consequent chronic immune induction is associated with a reduced SC clearance. As a result, this continuous response perpetuates a vicious circle that fuels inflammaging. Beyond immune cells and tissue cells, also adult stem cells from aged humans are affected (mesenchymal stem cells included). This evidence suggests that a senescent milieu could also reduce the stemness properties [22] or the differentiation capacity [23]. At the same time, these discoveries underline the specific actions and different features of SASP in the various tissues. However, the characterization of SASP is not complete yet: (i) what type of components are released like proteins (cytokines, chemokines, tissue remodelling factors, etc.), RNA, or DNA within or outside of extracellular vesicles, (ii) which are the SASP components derived from different cell types when senescent (fibroblasts, epithelial cells, endothelial cells, etc.), and finally (iii) which are the biological functions of various *secretomes* associated with senescence in different tissue microenvironments.

Altogether, this evidence suggests that inflammaging, sustained and perpetuated by a plethora of exogenous and endogenous stressors, is promoted by the accumulation of SCs during aging and perpetuated by both SASP spreading at systemic level and the SASP-associated bystander effects [24]. Importantly, SCs are abundant at all sites of ARDs, including not only malignancies but also degenerative disorders (above mentioned), suggesting that chronic inflammation induced by SCs might be a main driver of these pathologies [25, 26]. Accordingly, we recently investigated the “senescence” of endothelial cells (ECs) and the consequent endothelial dysfunction as one of the main trigger involved not only in the onset and progression of CVDs but also of other ARDs like osteoporosis [24, 27], since ECs are components of the stroma of all tissues and organs [28–30].

The abovementioned observations, about the clearing of senescent cells (SCs) from mice and its capacity to prolong their lifespan and healthspan, have opened a new era in the field of geroscience, with the creation of a new pharmacological branch devoted to *senotherapeutics*, that is, drugs able to affect (in a wide sense) the senescence process. Senotherapeutics currently includes three therapeutic approaches: (i) molecules able to selectively kill SCs, that is, *senolytics*; (ii) compounds with the capacity to attenuate the proinflammatory program of SCs, that is, SASP suppressors, or that modify the senescent phenotype, that is, senomorphics; and (iii) prevention of the accumulation of senescent cells. The latter is probably the “oldest” approach, since a plethora of antioxidants have been shown to delay the senescence process *in vitro* [31]. However, these findings have been hardly translated in promising findings, *in vivo* models, since no “usual” antioxidant (e.g., C and E vitamins) consistently increases lifespan or healthspan in mice models [31]. Moreover, data derived from human cohorts further suggest the inability of such compounds to prevent the major ARDs, even if consistent differences can be observed depending on the molecule tested [32]. Interesting preclinical findings have been reported for both senolytics and SASP-suppressing compounds. The development of senolytics has emerged thanks to both wide pharmacological screenings and differential gene expression studies. In addition, it has been optimized to identify *survival pathways* exploited by senescent cells to survive the proinflammatory milieu, where *they live in* (i.e., the SASP) [15, 33]. At present, five major senescent cell anti-apoptotic-pathways *(SCAPs)* have been identified and successfully targeted, that is, Bcl-family proteins, PI3K-Akt, p53, ephrin-tyrosine kinases, HIF-1*α*, and HSP90 pathways [33, 34]. The field is rapidly expanding, and the information provided by new technologies, for example, single-cell RNAseq, is expected to provide more insights into the heterogeneity of SCs in order to facilitate the development of new senolytic drugs [35]. Interestingly, different pathological phenotypes associated with aging have been targeted with various senolytics in mice models, that is, atherosclerosis, osteoporosis, and osteoarthritis. On the other side, a valuable alternative is represented by SASP-suppressing drugs. The major antiaging molecules, with accepted lifespan and healthspan promoting activity in mice, have been found to suppress the SASP, that is, rapamycin and metformin. Precisely, the activity of rapamycin has been explained through the well-known role of mTOR pathway in regulating SASP-factor secretion, while the mechanisms underlying the metformin's action on the SASP are until now unclear. However, both molecules are increasingly recognized as broad anti-inflammatory compounds. Another class of molecules with peculiar SASP-suppressing activity is JAK inhibitor, with interesting results obtained in terms of alleviation of frailty symptoms and amelioration of dysglycemia in old mice [36]. From a pharmacological point of view, senolytics have the advantage that noncontinuous, intermittent treatment appears as sufficient to produce a tangible effect, while with SASP suppressors, long-term treatment is often needed [33, 34]. However, at present, most of the drugs successfully tested in mice are compounds with an unfavourable profile of toxicity in humans (e.g., chemotherapeutics and immunosuppressors). Thus, their translation to clinical trial testing should be limited to specific situations, while a drug appropriate for a whole-population use is far to be discovered. At present, metformin appears as the most likely candidate for such use.

Preclinical data have uncovered a potential role for SCs in the promotion of a wide range of ARDs. Consistently, senolytics treatment has been associated with a plethora of beneficial effects: (1) improved cardiac ejection fraction in old mice; (2) enhanced vascular reactivity in old mice; (3) decreased vascular calcification, increased vascular reactivity, and reduced senescence burden in the plaque of apoE^−/−^ mice; (4) decreased frailty, osteoporosis, and loss of intervertebral disc glycosaminoglycans in progeroid mice; (5) decreased gait disturbance in mice after radiation damage to a leg; (6) attenuated haematological dysfunction caused by whole body radiation; (7) increased coat density; and (8) improved pulmonary function and reduced pulmonary fibrosis in mice with bleomycin-induced lung damage [33, 34]. These observations are consistent with the hypothesis that SCs drive organism aging. Thus, their targeting must benefit more than one age-related phenotype, counteracting aging itself as a whole rather than one ARD at a time.

As mentioned above, newly discovered senolytics molecules are drugs already present in the market and used for the treatment of a wide range of life-threatening diseases, for example, various types of cancer. Thus, they are often accompanied by a range of potential side effects. This implies that the repurposing (or the extension of clinical indications) of these drugs should be limited to conditions where the possible benefits overcome the potential risks. Kirkland and colleagues have proposed a precise hierarchy of possible clinical settings, where to test such compounds [34]. Potential clinical trials are the following: (1) simultaneous attenuation of multimorbidity; (2) alleviation of potentially fatal diseases, for example, pulmonary fibrosis; (3) treatment of conditions with localized senescent cell accumulation, for example, osteoarthritis, possibly through local delivery of the drug to limit systemic toxicity; (4) treatment of conditions characterized by accelerated aging, for example, patients exposed to radio- and chemotherapy, HIV-infected patients, and progeroid syndromes; (5) increasing physiological resilience, that is, the capacity to recover after a stress, in the case that the stressor is known to promote the accumulation of senescent cells; and (6) alleviation of frailty [34]. The discoverers of senolytics have announced their intention to test these molecules in humans in specific setting in the next future [37]. However, at present time, the only scheduled antiaging trial is the Targeting Aging with Metformin (TAME) trial [38]. Despite the lack of a univocal mechanism of action for this drug, the evidence of a cardioprotective, cancer-preventive, and lifespan/healthspan-promoting activity for metformin is strong enough to justify an extension of its use to nondiabetic subjects. Data from human cohorts have clearly disclosed a marked anti-inflammatory effect of this compound, rendering metformin the ideal candidate to counteract inflammaging [38–40]. In addition, its favourable toxicological profile surely supports a “widespread” use of this drug, since the only serious adverse effect, that is, lactic acidosis, has an incidence of less than 10 episodes per 100,000 person-year (and it affects mainly people with renal impairment). The TAME trial will involve 3000 patients with at least one ARD, aged 65–79, and will measure a composite outcome that includes cardiovascular events, cancer incidence, dementia, and mortality. Functional and geriatric endpoints will be assessed too. While waiting for the results, it is worth mentioning that this trial represents the first case of intent to treat aging as a disease. In the case of positive results, this trial will pave the way for a revolution in the management of elderly subjects, with preventive medicine able to postpone ARD development instead of punctual medicine treating each disorder as a stand-alone entity.

In this issue, a large range of current directions in research about this topic will be described and discussed. Thus, we think that the papers of this issue could be of interest to the readers of this journal.

The group of E. Dozio et al. investigated, for the first time, the role of vitamin D status in patients with acute aortic dissection (AAD), since evidence sustains an inverse association between serum 25-hydroxy vitamin D (25OHD) levels and the onset of several cardiovascular conditions, such as aortic aneurysms and thoracic aortic dilatations. Interestingly, a condition of hypovitaminosis D associated with an increased osteocalcin levels was observed in the study population. Thus, these molecules may be helpful to identify individuals at high risk to develop AAD as well as to study preventing strategies.

The importance of 25OHD, in maintaining of healthy status of all our body's systems with advancing age, is also emphasized by L. Elizondo-Montemayor and coworkers. Precisely, they performed a longitudinal 12-month follow-up study in order to determine the vitamin D seasonal changes and their association with anthropometric parameters, lifestyle factors, and proinflammatory cytokines in an older adult Mexican population. Their results show a great prevalence of vitamin D deficiency and insufficiency across all seasons, with significantly greater prevalence of deficiency in winter compared with summer and autumn. Vitamin D levels were negatively correlated with BMI, waist circumference, and weight, as well as with gender differences and TNF-*α* levels. While WC explained almost half of the variations in vitamin D levels in women, BMI was the second significant predictor of vitamin D. However, neither dietary vitamin D intake nor sun exposure affected 25OHD levels.

The group of E. Dozio et al. also evidenced, in a cross-sectional study, that diabetic patients, with chronic kidney disease complication on dialysis (CKD-G5D), show significant increased levels of soluble receptor for advanced glycation end products (AGEs; sRAGE). Thus, they concluded that sRAGE may be a marker of cardiac remodeling. Indeed, its increase could be a potential protective mechanism against the increased risk of cardiovascular complications related to AGEs and inflammation.

According to data above described, a dramatic increase of CVDs characterizes Western populations, because of aging population phenomenon. The group of L. Iop et al. summarizes the intrinsic and extrinsic causes related to cellular vascular senescence and their role in the onset of cardiovascular pathologies. Additionally, they dissect the effects of aging on the cardiac endogenous and exogenous reservoirs of stem cells. Finally, they offer an overview on the strategies of regenerative medicine that have been advanced in the quest for heart rejuvenation.

Among the exogenous risk factors linked to cellular vascular senescence, the diet represents an emergent CVD inductor. The group of M. Malavolta et al. examined, in a research study, the effects of postprandial sera derived from healthy adults and elderly volunteers who consumed meat meals on human coronary artery endothelial cell (HCAEC) oxidative stress, gene expression, DNA damage, and cellular senescence. They observed that a single exposure to postprandial serum induces a slight increase in ROS that is associated with modulation of gene expression pathways related to oxidative stress response and metabolism. The postprandial-induced increase in ROS is not associated with a measurable DNA oxidative damage. However, repeated exposure to postprandial serum induces an acceleration of cellular senescence. Taking into account the deleterious role of cellular senescence in ARDs, the results suggest a new mechanism by which excessive meat consumption and time spent in postprandial state may affect health status during aging.

The above-described effects are mediated through epigenetic factors. The group of A. Giuliani et al. focused its attention on the deep reshaping of microRNA expression and modulation of mitochondria activity, both master regulators of the SASP in tissue aging. In particular, they propose a network linking nuclear-encoded SA-miRNAs to mitochondrial gene regulation and function in aging cells. In this conceptual structure, SA-miRNAs can translocate to mitochondria (SA-mitomiRs) and may affect the energetic, oxidative, and inflammatory status of senescent cells. They discuss the potential role of several of SA-mitomiRs (i.e., let-7b, miR-1, miR-130a-3p, miR-133a, miR-146a-5p, miR-181c-5p, and miR-378-5p), using miR-146a as a proof-of-principle model. Finally, they suggest a comprehensive, metabolic, and epigenetic view of the senescence process, in order to amplify the range of possible approaches to target inflammaging, with the ultimate goal of decelerating the aging rate, postponing or blunting the development of age-related diseases.

The onset of ARDs, CVDs included, also is significantly linked to an impaired immune system (as mentioned above), which shows in old ages several changes, defined as immunosenescence. At present, all molecular and cellular mechanisms involved still remain to be identified. However, the human immunodeficiency virus (HIV) infection and the consequent acquired immune deficiency syndrome (AIDS) represent an optimal model for studying immunosenescence. T. Sokoya and coworkers describe in a review the role of systemic immune activation in the immunopathogenesis of HIV infection, its causes and the clinical implications linked to immunosenescence in adults, and the therapeutic interventions that have been investigated.

Furthermore, the group of F. Sizzano et al. from Nestlè Institute of Health Sciences investigated for the first time the response of the main lymphocyte subsets to the induced oxidative stress in semi-super-centenarians (CENT), their offspring (OFF), elderly 52 controls (CTRL), and young individuals (YO), by using flow cytometry. Results showed that the ratio of the ROS levels between the induced and noninduced (I/NI) oxidative stress conditions was higher in CTRL and OFF than in CENT and YO, in almost all T, B, and NK subsets. Moreover, the ratio of reduced glutathione levels between I/NI conditions was higher in OFF and CENT compared to the other groups in almost all the subsets. Finally, they observed significant correlations between the response to the induced oxidative stress and the degree of methylation in specific genes on the oxidative stress pathway. Globally, these data suggest that the capability to buffer dynamic changes in the oxidative environment could be a hallmark of longevity in humans.

According to immunosenescence evidence, the group of E. Costantini et al. reports in a review the link between immune and nervous system and how the immunosenescence and inflammaging can contribute to neurodegenerative diseases.

Changes in endocrine system also characterize old people and are significantly associated with ARD onset. In particular, thyroid dysfunction and its impact on cognition in older individuals have been demonstrated, even if some aspects remain unclear. The group of G. Traina et al. investigated the changes which accompany mouse thyroid gland in old age. The results obtained evidence changes in the height of the thyrocytes and in the amplitude of interfollicular spaces, anomalous expression/localization of thyrotropin, thyrotropin receptor, and thyroglobulin aging. Thyrotropin and thyrotropin receptor are upregulated and are distributed inside the colloid while thyroglobulin fills the interfollicular spaces. Furthermore, they found a higher expression of galectin-3 and a delocalization of neutral sphingomyelinase in the thyroid of old animals.

In order to counteract ARD onset and complications, several anti-inflammaging/anti-ARD treatments are emerging, ranging from pharmacological targeting of aging, basic biological assays, and big data analysis to the recent use of young blood, stem cells, cellular, genetic, and epigenetic reprogramming, or other techniques of regenerative medicine. C. R. Balistreri summarizes them in a review, stressing that only a little fraction of these approaches have the features for being tested in clinical applications. Thus, she describes new emerging molecules, drugs, and procedures, by evidencing potential benefits and limitations.

Among these approaches, the use of senolytics molecules emerges. Accordingly, the group of M. Malavolta et al. also summarizes in a review the i*n vitro* and *in vivo* effects of fifteen Nrf2- (nuclear factor erythroid-derived 2-related factor 2 pathway-) interacting natural compounds (tocotrienols, curcumin, epigallocatechin gallate, quercetin, genistein, resveratrol, silybin, phenethyl isothiocyanate, sulforaphane, triptolide, allicin, berberine, piperlongumine, fisetin, and phloretin) on cellular senescence and discusses their use in adjuvant cancer therapy.

*Fabiola Olivieri* *Francesco Prattichizzo* *Johannes Grillari* *Carmela R. Balistreri*
